# Attitudes to and experience of disease management programs in primary care—an exploratory survey of general practitioners in Germany

**DOI:** 10.1007/s10354-021-00867-1

**Published:** 2021-08-02

**Authors:** Julian Wangler, Michael Jansky

**Affiliations:** Centre for General and Geriatric Medicine, University Medical Centre Mainz, Am Pulverturm 13, 55131 Mainz, Germany

**Keywords:** Disease management programmes, General practitioner, Chronic diseases, Multimorbidity, Disease-Management-Programme, Hausarzt, Chronische Erkrankungen, Multimorbidität

## Abstract

**Background:**

Disease management programs (DMPs) were set up in Germany in 2003 to improve outpatient care of chronically ill patients. The present study looks at the attitudes and experiences of general practitioners (GPs) in relation to DMPs, how they rate them almost 20 years after their introduction and where they see a need for improvement.

**Methods:**

A total of 1504 GPs in the Federal States of Rhineland Palatinate, Saarland and Hesse were surveyed between December 2019 and March 2020 using a written questionnaire.

**Results:**

In total, 58% of respondents rate DMPs positively and regard them as making a useful contribution to primary care. The guarantee of regular, structured patient care and greater compliance are regarded as particularly positive aspects. It was also established that diagnostic and therapeutic knowledge was expanded through participation in DMPs. 57% essentially follow the DMP recommendations for (drug) treatment. Despite positive experiences of DMPs in patient care, the GPs surveyed mention various challenges (documentation requirements, frequent changes to the programmes, inflexibility). Univariant linear regression analysis revealed factors influencing the satisfaction with DMPs, such as improvement of compliance and clearly defined procedures in medical care.

**Conclusion:**

Most of the GPs surveyed consider the combination of continuous patient care and evidence-based diagnosis and treatment to be a great advantage. To better adapt DMPs to the conditions of primary care, it makes sense to simplify the documentation requirements, to regulate cooperation with other healthcare levels more clearly and to give GPs more decision-making flexibility. Increased inclusion of GP experience in the process of developing and refining DMPs can be helpful.

**Supplementary Information:**

The online version of this article (10.1007/s10354-021-00867-1) contains supplementary material, which is available to authorized users.

## Abstract (de)

**Hintergrund:**

Im Jahr 2003 wurden in Deutschland die Disease-Management-Programme (DMP) zur Verbesserung der Versorgung chronisch kranker Patienten im ambulanten Bereich eingerichtet. Die Studie geht der Frage nach, welche Einstellungs- und Erfahrungswerte Hausärzte in Bezug auf DMP vertreten, wie sie diese rund 2 Dekaden nach ihrer Einführung bilanzieren und wo sie Verbesserungsbedarfe ausmachen.

**Methoden:**

Mittels schriftlicher Befragung wurden zwischen Dezember 2019 und März 2020 insgesamt 1504 Hausärzte in den Bundesländern Rheinland-Pfalz, Saarland und Hessen befragt.

**Ergebnisse:**

Von den Befragten beurteilen 58 % die DMP positiv und erachten sie als nützlichen Beitrag zur hausärztlichen Versorgung. Besonders positiv wird die Sicherstellung einer regelmäßigen, strukturierten Patientenbetreuung und die Verbesserung der Compliance gesehen. Ebenfalls wird konstatiert, dass die diagnostischen und therapeutischen Kenntnisse durch die DMP-Teilnahme erweitert werden konnten. Prinzipiell richten sich 57 % nach den DMP-Empfehlungen zur (medikamentösen) Therapie. Trotz positiver Erfahrungen mit DMP in der Patientenversorgung benennen die befragten Hausärzte verschiedene Herausforderungen (Dokumentationspflichten, organisatorische Veränderungen, Starrheit des DMP-Konzepts). Die Ergebnisse einer univariaten linearen Regression zeigen mehrere Einflussfaktoren für die Beurteilung von und Zufriedenheit mit DMP, darunter die Verbesserung der Compliance und die klare Regelung von Versorgungsabläufen.

**Schlussfolgerung:**

Die Kombination aus kontinuierlicher Patientenbetreuung und diagnostischer sowie therapeutischer Evidenzorientierung wird von den befragten Hausärzten als großer Vorzug erachtet. Um DMP in Zukunft noch hausarztkonformer zu gestalten, erscheint es sinnvoll, den Dokumentationsaufwand zu vereinfachen, die Zusammenarbeit mit anderen Versorgungsebenen klarer zu regeln und Hausärzten mehr Entscheidungsflexibilität einzuräumen. Ein verstärkter Einbezug von hausärztlichen Erfahrungen im Prozess der DMP-Weiterentwicklung kann bei dieser Optimierung hilfreich sein.

## Introduction

Establishing healthcare structures to provide better diagnosis, treatment and prevention of chronic diseases is a major challenge within the healthcare system [1–3]. In order to make healthcare more effective and efficient, disease management programs (DMPs) were established in Germany in 2003 as statutory treatment programmes in the outpatient sector, especially with a focus on primary care [4, 5]. Meanwhile, there are now more than 8 million health insurance holders enrolled in the existing DMPs, 1.2 million of whom are enrolled in more than one program [4]. DMPs aim to better structure treatment processes and are based on current medical knowledge as well as evidence-based guidelines with regard to specifications for diagnostics and therapy [6]. In addition, the intention is to strengthen the collaboration between the healthcare levels, for example by means of statutory job descriptions and therapy descriptions, and fixed check-up intervals [7–9]. Consistent recording of all examination and treatment results serves to coordinate individual healthcare steps, so that unnecessary duplication of investigations or examinations can be avoided.

Alongside the regular care of enrolled patients, doctors who participate in DMPs receive mandatory training courses. Moreover, medical practices that offer treatment within the framework of DMPs must fulfil set quality requirements; this can lead to changes in the practice’s workflow [10, 11]. Depending upon the program, structured training courses are also offered to patients in support of their treatment and/or for the purposes of prevention. A uniform electronic documentation system provides ongoing evaluation and quality assurance. To this end, treatment data are recorded centrally and the achieved treatment progress is fed back to participating doctors [4, 12, 13].

It has been found that patients enrolled in DMPs are better informed about their disease and the associated risks, and display greater treatment compliance [8, 9, 14]. However, in terms of the demonstrable effects of DMPs, there are currently very few reliable efficacy studies available for the German healthcare context. In most cases, an efficacy control is not readily possible based on the legally prescribed documentation, since there is no control group [15]. Moreover, there are unknown disturbance variables, which can only be neutralised by strict randomisation [1, 14, 45].

Several studies indicate favourable effects on mortality and process parameters for the type 2 diabetes DMP [12, 16–22]. A multicentre but non-randomised cross-sectional study recently examined the benefit of the bronchial asthma and COPD DMPs. However, the authors were unable to prove any clinically relevant advantages for DMP participants, either in terms of disease control or quality of life [23]. Despite the methodological limitations, an initial analysis of the effectiveness of the CHD DMP indicates positive trends in terms of mortality, cost development and guideline-based prescribing [24]. Clinically randomised studies conducted in other countries have already demonstrated the beneficial effects of comparable programs [18, 25–28].

The DMP objectives cannot be achieved without the substantial participation of general practitioners as primary care providers with access to a broad, unselected patient base [10, 11, 22]. In this respect, general practitioners play a key role in the recruitment of patients, ensuring compliance and coordination of the treatment process [7, 22].

Since the introduction of DMPs, there has been a controversial debate among general practitioners about the value and benefit of the structured treatment programs [29–32]. One group emphasises their beneficial potential (diagnostic and therapeutic accuracy, more evidence-based practice, transparency of decision-making processes, more efficient use of resources), while another complains about excessive impacts on primary care (strict provisions that preclude individual patient care, changing of routine workflows, excessive documentation requirements) [33].

Despite the important role that primary care plays in the DMP concept, empirical studies have only looked at it sporadically; there is a lack of up-to-date findings. In particular, there is hardly any reliable information available concerning questions of acceptance, satisfaction and the associated attitudes to and experience of DMPs in everyday primary care, especially in German-speaking countries. For example, a survey of 752 non-systematically recruited GPs shows that DMPs are judged better by primary care physicians today than shortly after their introduction. The more consistent and continuous care of chronically ill patients is seen as a clear advantage [34]. Almost 20 years after introduction of the structured treatment programs, the present study aims to assess them from the general practitioner’s point of view.

### Research interest

In order to obtain an up-to-date and broad picture of GPs’ attitudes to and experience of DMPs, a written survey was conducted among German GPs between December 2019 and March 2020. The study addressed the following questions:

- Which DMP programs are GPs participating in?
- What attitudes do general practitioners have towards DMPs?
- What experiences have they had in patient care?
- How do they rate the concrete benefit of DMPs?
- What improvements would they like to see?

## Materials and methods

### Study design

The written, anonymised survey is based on a preliminary study [34], in which the questionnaire design was tested. Since, especially in German-speaking countries, there is still a lack of studies addressing aspects of acceptance and application of DMPs by (outpatient care) doctors, the study was updated and repeated on a much larger scale, in order to check the extent to which the previous results could be confirmed.

### Survey method

The questionnaire is based on a review of the state of research, taking particular account of studies carried out to date into the general practitioner’s view of DMPs and/or their acceptance [6, 35–37]. In addition, preliminary discussions on the topic were held with a total of ten GPs. These were decisive for specification of the questionnaire and helped in the development of the item batteries that were used (questions 4, 15, Supplementary Information). Alongside the standardised questions, several open questions were included to ensure an explorative approach that was open to new aspects. A pretest was conducted prior to application in the field.

The questionnaire (approximate completion time 10 min) consists of four blocks: attitudes to and positions on DMPs, participation in individual DMPs and evaluation, assessment and inventory of effects, prospective aspects and optimisation potentials.

### Recruitment and participants

All 6562 general practitioners actively working as clinicians in Rhineland-Palatinate, Saarland and Hesse were invited to participate in the anonymised survey by means of a postal letter. As well as filling in the written questionnaire, they had the alternative option of answering the survey online; it was loaded onto the survey portal of the implementing department as a LimeSurvey (Lime Survey GmbH, Hamburg, Germany) questionnaire. The sociodemographic features of age, gender, practice location, type of practice and patients per quarter were collected.

### Data analysis

The data were analysed using SPSS 23.0 for Windows (IBM Corp., Armonk, NY, USA). Alongside the descriptive analysis, a factor analysis (varimax rotation) was performed to obtain more accurate information about the extent to which certain views of DMPs correspond to each other. In order to check the prerequisite for a factor analysis (see Table 1), the Kaiser–Meyer–Olkin sampling adequacy of the random sample was first of all tested and found to be particularly good in the present case (0.951). Secondly, we carried out Bartlett’s test of sphericity to check the hypothesis that all correlation variables have a value of zero in the basic population. A significant result, as in the present case, allows the interpretation that in the basic population, “there are correlations at least between some variables; the null hypothesis can therefore be rejected” [38, p. 325]. In the case of all included variables, the commonalities are also significantly above the standard threshold of 0.5, so that each individual item variable is suitable for the factor analysis. In order to determine the exact number of factors, in addition to considering the Kaiser criterion, the scree test was used. The scree test is a visual test that looks for disjunctures in the pattern of eigenvalues as a function of factor succession.

**Table 1:** General practitioners’ attitudes to disease management programs (DMPs). Question: *Which of the following statements do you agree with?* (*N* = 1504; response categories *Completely agree/mostly agree* combined and rotated component matrix)

		Rotated component matrix		
*Disease management programs have …*	Overall agreement (%)	Comp. 1 (explained variation: 41.9%)	Comp. 2 (explained variation: 15.1%)	Comp. 3 (explained variation: 8.2%)
Helped to ensure that chronically ill patients are cared for proactively and continuously	73	0.693	−0.345	0.349
Improved compliance of chronically ill patients	73	0.698	−0.349	0.283
Brought about a change in workflows/responsibilities within the practice	72	0.242	0.095	0.830
Helped to ensure that patients are increasingly treated on the basis of evidence-based medicine/guidelines	71	0.709	−0.165	0.220
Resulted in a great deal of unnecessary bureaucracy and/or documentation work	71	−0.684	0.416	0.406
Strengthened the position of GPs in the care of chronically ill patients	63	0.711	−0.317	0.185
Led to the successful management of multimorbid/chronically ill patients	63	0.815	−0.282	0.281
Resulted in a clearly defined procedure, thereby increasing transparency of decision-making and ensuring the safety of medical action	54	0.706	−0.183	0.324
Helped to prevent over- and/or undertreatment	49	0.736	−0.180	0.117
Increased the dependency of GPs upon health insurers	46	−0.289	0.603	0.244
Improved the diagnostic and therapeutic safety of GPs	45	0.783	−0.086	0.178
Not significantly changed the quality of care for chronically ill patients	43	0.358	0.450	−0.104
Led to more efficient patient care	40	0.789	−0.160	0.057
Reduced costs for the healthcare system	32	0.756	−0.047	0.080
Effectively improved the collaboration between GPs and consultants in the care of chronic diseases	32	0.714	0.061	−0.175
Restricted the therapeutic freedom of GPs	28	−0.148	0.832	−0.001
Resulted in patients being treated and considered less individually	22	−0.081	0.842	−0.104
Involved a heavy organisational/logistical burden for the practice managers	17	−0.177	0.126	0.698

Extraction method: principal component analysis

Rotation method: varimax, Kaiser normalisation

Rotation converged in five iterations

Explained overall variance: 65.2%

Kaiser–Meyer–Olkin sampling adequacy: 0.951

Bartlett significance: *p* < 0.001

A univariant linear regression analysis (*p* < 0.05) was used to identify possible influencing factors [38]. Univariate linear regression focuses on determining relationships between one independent (explanatory variable) variable and one dependent variable. In the analysis, all factors were considered which, according to Cohen [40], have at least a slight explained variation (| R^2^ | = 0.02).

Evaluation of the open questions was carried out by both authors and is based on recoding in accordance with Mayring qualitative content analysis using MAXQDA software (VERBI, Berlin, Germany) [39]. A system of categories was determined during examination of the responses and this was repeatedly checked and modified, if necessary, as evaluation progressed. In this way, it was possible to condense and systematise differences and commonalities in the data in the form of argumentation and/or problematisation patterns.

## Results

### Random sample

Out of a total of 1556 returned questionnaires, 1504 fully completed questionnaires were included in the evaluation. The response rate was 23%, measured against the total number of doctors contacted. The random sample is structured as follows:

- Gender: 52% male, 48% female
- Practice location: 45% in medium-sized towns and cities, 55% in small towns and rural areas
- Type of practice: 55% single-handed practices, 42% group practices, 3% other
- Patients per quarter: 18% < 1000, 29% 1000–1500, 53% > 1500
- Average age: 55 years (median: 56)

### Attitudes to and positions on DMPs

While 58% of respondents consider DMPs to be a positive element in medical care, 36% express scepticism and/or rejection (6% undecided). At a figure of 57%, the majority also say that, based on their own assessment and experience, DMPs had been of very great (14%) or fairly great (43%) benefit for patient care (27% fairly small benefit, 11% no benefit, 5% difficult to say). 37% of respondents report that their basic attitude to DMPs had significantly (15%) or slightly (22%) improved over the past few years, in 46% it has remained the same, 17% report a moderate (11%) or significant (6%) change for the worse. 43% of doctors, especially those in more rural practices, report that they have developed a greater appreciation for DMPs in recent years; among doctors in urban areas, this figure is 31% (*p* < 0.001).

From the respondents’ point of view, the advantages of DMPs for primary care clearly predominate (see Table 1, Overall agreement). A factor analysis was performed to obtain more accurate information about the extent to which certain views of DMPs correspond to each other.

The aim of the factor analysis is to condense a larger number of variables into factors based on systematic relationships (correlations). By condensing the variation of a plurality of variables into a much smaller number of common factors (data reduction), we tried to discover underlying common dimensions. The varimax method that was chosen for this is the most frequently used method for arriving at interpretable factorial solutions. As described in the Materials and methods section, the statistical requirements for performing the factor analysis were met.

The analysis turned out in favour of a three-factor solution, since in the present case, three factors have a disproportionately high explanatory power and in each case an eigenvalue > 1 (Kaiser criterion). In addition to this, the explained overall variance is comparatively high (65%) in a three-factor solution. Even according to the scree test, the pattern of eigenvalues most readily points to a three-factor solution. Consequently, such a structure appears to be plausible and stable. The value of 0.4/−0.4 was chosen as the limit beyond which an item loads onto a factor [37].

In keeping with the outlined procedure, it is possible to distinguish between three clusters of GPs. The largest group notably reports perceptible progress in diagnosis, monitoring and treatment, as well as compliance effects. The stricter alignment with guidelines is perceived as a distinct advantage. Overall, a strengthening of the GP’s role is perceived. Cluster two stresses perceived negative aspects, including increased dependence on the health insurance funds or the restriction of a doctor’s therapeutic freedom. The third cluster focuses on adaptations within the practice to comply with DMP requirements.

All three groups complain about the amount of time and effort spent on documentation; overall, three quarters of all doctors point this out. Moreover, only a few of the respondents perceive an improvement in collaboration with specialist colleagues as a result of participation in one or more programmes. The doctors are doubtful about any lasting efficiency benefits in patient care due to DMPs.

### DMP participation and rating

A total of 90% of respondents are currently participating in one (38%) or more (52%) DMPs; a further 5% have previously participated (5% no current or previous participation). Most of the current participants are involved in the type 2 diabetes DMP (87%), followed by the CHD DMP (86%). These are followed by the DMP for COPD (82%) and bronchial asthma (80%). 21% are participating in the DMP for type 1 diabetes, which can be explained by the specific preconditions of this program.

Based on the experience of the respondents, often covering many years, the DMP for type 2 diabetes has the best rating (39% very good, 39% fairly good). The Type 1 diabetes DMP is likewise well received by the 310 respondents participating in it (31% very good, 38% fairly good). These are followed by the DMP for CHD (23% very good, 41% fairly good), COPD (16% very good, 43% fairly good) and bronchial asthma (12% very good, 41% fairly good).

There appears to be a varying degree of acceptance of the different DMP components. For example, 85% judge the regular recall of patients, as envisaged in the intervals currently prescribed by the DMP, to be very beneficial or fairly beneficial. 73% appreciate the patient training courses that are offered in support of their treatment. 60% consider the mandatory training courses for doctors to be a very good or fairly good thing. In contrast, only 36% are satisfied with the external recording of the treatment and 32% with the current design of the documentation.

### Positive and negative experiences in daily practice

1103 doctors completed the open questions provided. In the course of encoding, a number of recurring argumentation and problematisation patterns emerged. In their own words, the respondents highlight the regular patient care, the structure in patient management (therapy adherence) and the ongoing treatment monitoring as positive. Likewise, a large proportion of respondents appear to be satisfied with a better knowledge of the guidelines and the structured opportunity to attend targeted training courses.

In addition to the high amount of documentation required, difficulties in the administrative process are problematized. For example, patients who missed an appointment once are immediately removed from the program and a great amount of effort is required to re-enrol them. Other complaints were the delayed response about the participation status of patients or the fact that evaluation reports and feedback reports are submitted very late. Also, the often-unpredictable adjustment of the framework conditions of the programs impede the workflow (e.g. changing requirements and forms, changes in IT systems). Another object of criticism is the perceived lack of flexibility of the DMP design, which allegedly leaves GPs too little situational freedom of action (e.g. recall intervals, prescribing and treatment guidelines). The fact that an adequately functioning interface with other healthcare levels, in particular outpatient consultants, has not developed in pace with the DMP structures and guidelines is experienced as a huge problem. Part of the respondents criticize that health insurers exert increased pressure on patients to take part in DMPs, thereby often forcing GPs to participate in the programmes. Also, many respondents are of the opinion that patients enrolled in DMPs were not well enough informed or motivated over the longer term. Other criticisms relate to the fee structure, which many respondents think it is not proportionate to the amount of effort and extra burden in daily practice and training courses that are not always practically oriented and suited to the level of knowledge of the doctors.

### Assessment and inventory of effects

Implementation of the DMP often requires a change to working practices, routines and allocation of duties in the practice. For example, 79% of respondents report that they have trained one member of their own practice staff (19%), or even several people (52%) through to the entire staff (8%), once or several times in connection with DMP participation. Irrespective of this, the changes in the everyday running of the practice caused by DMPs can, under certain circumstances, result in delays or other difficulties. Around half of the respondents participating in at least one DMP (50%) report having encountered obstacles and/or complications in their everyday practice frequently (14%) or occasionally (36%; 32% rarely, 18% never).

Despite such temporary problems and adjustments, the rest of the results indicate that the survey participants considered their involvement to be a positive thing, when they looked back. At 51%, a majority report that the treatment of the enrolled patients benefited very much (8%) or quite a lot (43%) from the DMP (30% not so much, 14% not at all, 6% it differs, difficult to say). Doctors who trained at least half their staff in the course of DMP participation are much more likely to report that the treatment of patients benefited from the DMP (61%) than doctors who only trained a few members of their staff or none all (44%; *p* < 0.001).

On the basis of an item set, DMP participation is clearly rated as positive overall. It is clear that the majority of respondents accept complications and extra work resulting from program participation, because they believe these are outweighed by the benefits (see Table 2). It also emerges that, in the view of the GPs, DMP participation has favourable consequences in terms of diagnostic and therapeutic procedures.

**Table 2:** Inventory of disease management program (DMP) participation. Question: *Based on your own experience of DMPs, which of the following statements do you agree with?* (*N* = 1426)

	Completely agree/largely agree (%)	Largely disagree/completely disagree (%)
“The advantages of disease management programmes outweigh the disadvantages and difficulties.”	59	41
“I essentially follow the DMP recommendations for (drug) treatment.”	57	43
“I have improved my own skills as a result of participating in disease management programs.”	49	51
“I can hardly imagine doing without disease management programs in my practice.”	48	52
“I have learnt something new about diagnosis and/or treatment through participating in disease management programs.”	44	56

### Factors influencing the rating of and satisfaction with DMPs

The results of a univariant linear regression analysis reveal a series of stronger and weaker influencing factors for key dependent variables (see Table 3). As expected, the things that particularly stand out in the basic assessment of structured healthcare programmes by the respondents are positive compliance experiences (35% of overall variance, R^2^) and successes in the consequent treatment of chronically ill patients (47% of overall variance, R^2^). Increases in efficiency (30% of overall variance, R^2^) and improvement of individual diagnostic skills (28% of overall variance, R^2^) are also important reasons for the respondents giving a positive opinion of DMPs.

**Table 3:** General practitioners’ perception of disease management programs (DMP): univariant linear regression, identified influencing factors^a^ (*N* = 1504)

*Dependent variable: perceived benefit of DMPs* *(“In your opinion or experience, how great is the overall benefit of DMPs for patient care?”)*							
Independent variable: (possible influencing factor and/or predictor)	R^2^	R^2^ corrected	F (df = 1; 1502)	Regression coefficient β	Significance	95% confidence interval	Standard error
Improvement in compliance (question 4)	0.346	0.346	795.916	0.544	0.000	0.506; 0.581	0.019
Restriction of therapeutic freedom (question 4)	0.083	0.083	136.643	−0.233	0.000	−272; −0.194	0.02
Improvement in collaboration with consultants (question 4)	0.130	0.129	224.074	0.289	0.000	0.251; 0.327	0.019
Strengthening the position of GPs within the healthcare process (question 4)	0.320	0.319	705.267	0.513	0.000	0.475; 0.551	0.019
Preventing over- and/or undertreatment (question 4)	0.253	0.253	509.801	0.405	0.000	0.369; 0.44	0.018
Clearly defined procedure in medical care (question 4)	0.272	0.271	559.857	0.418	0.000	0.383; 0.452	0.018
Proactive, continuous treatment (question 4)	0.405	0.405	1023.42	0.601	0.000	0.574; 0.638	0.019
Successful management of multimorbid/chronically ill patients (question 4)	0.472	0.472	1345.378	0.606	0.000	0.574; 0.638	0.017
More efficient patient care (question 4)	0.296	0.295	631.237	0.434	0.000	0.4; 0.468	0.017
Improvement of diagnostic skills (question 15)	0.284	0.284	596.341	0.439	0.000	0.404; 0.475	0.018

^a^ Listed are all factors that, according to Cohen [40], have at least a slight explained variation. Classification: slight/weak explained variation |R^2^| = 0.02; average/moderate explained variation = 0.13; high/strong explained variation |R^2^| = 0.26

### New programs, prospective aspects and optimisation potential

A significant proportion of respondents are open to participating in additional DMPs that are currently in the development or implementation phase. There is a particularly high level of interest in a heart failure DMP (33% intend to participate, 40% might consider it) followed by a chronic back pain DMP (24% intend to participate, 34% might consider it).

Evaluation of a further open question indicates that the majority of respondents want to see a substantial reduction in documentation requirements for DMP in the future (e.g. dispensing with re-enrolment forms), simplifying interactions with the DMP datacentre and more organisational continuity in the treatment programs. Other frequently mentioned aspects are allowing doctors more decision-making flexibility (e.g. regarding patient recall and treatment-related decisions) as well as strengthening and better structuring of communications and/or cooperation at the interfaces with other healthcare actors. Overall, according to many respondents, DMPs should be designed with an even lower threshold for doctors and patients, thereby enabling them to play an even greater role in the care of vulnerable groups. Moreover, training courses should be more customised, offered more widely, include practice staff more than has hitherto been the case and should be free, where possible. Last but not least, the participating doctors recommend a fee structure that reflects the amount of effort, possibly by the creation of more billing codes.

From the responses, it emerges that the role of GPs should be further strengthened within the DMP design. Ways of achieving this are seen in greater GP compliance, greater orientation towards everyday application and better coordination with primary healthcare guidelines. In the spirit of a bottom-up process, GPs should have the opportunity to contribute to improvements and adaptations of the programmes, based on experience and practice. Accordingly, in a follow-up question, 80% of participants state that it would be very important or fairly important for GPs to be more involved than before in the development of new or optimisation of existing DMPs.

## Discussion

### Principal findings and comparison with prior work

The survey of 1504 general practitioners in Germany shows that almost two decades after DMPs were introduced into everyday primary care, they have been widely adopted and largely accepted. The majority of respondents participate in more than one DMP. At the time of introducing the programs, fears predominated among many GPs with regard to the curtailment of treatment freedom and the loss of consideration for individual patient needs [17, 35]. Today, primary care doctors predominantly emphasise the added value and potential of DMPs when it comes to the diagnosis, monitoring and treatment of chronically ill and multimorbid patients. The survey confirms all major findings of the preliminary study, which already determined positive attitudes and experiences of general practitioners towards DMPs (especially with regard to the continuity, stability and evidence orientation of patient care). The findings also coincide with the results of an older study from the USA, showing that three quarters of the physicians believed that DMPs increased the overall quality of patient care and the quality of care for the targeted disease [41].

The results of the present study confirm impressions and statements from specialist medical discourse in recent years, indicating a gradual change in the position of general practitioners towards DMPs [42–45]. This presumably correlates with a changing landscape in terms of knowledge acquisition and advanced training, and an increasingly scientific orientation of general medicine. Nowadays, many GPs increasingly base their work on standardised, evidenced-based interventions, of which DMPs are a good example [43, 44].

Many GPs have come to value DMPs, because they ensure regular, structured patient care and are beneficial for guideline-oriented disease management. At the same time, DMPs can considerably improve patient management and compliance [6, 12, 17, 23]. Consequently, the majority of respondents believed that the establishment of DMPs had upgraded the role of GPs in the area of chronic diseases. Likewise, the majority stated that the treatment of the enrolled patients and their personal knowledge and skills had benefited from participation in DMPs. More than half the respondents now essentially comply with the DMP treatment recommendations, thereby exhibiting marked compliance with the guidelines—another finding that has changed relative to the first surveys conducted shortly after the programs had been introduced [29–33, 35]. The results also confirm that many GPs could not now imagine doing without DMPs in their everyday practice [34, 45].

Nevertheless, the survey results also indicate weaknesses. There are repeated complaints about the amount of bureaucratic effort required for documentation and patient (re)enrolment, as well as for communications with the DMP datacentre. Frustration at frequent changes to the programs is also reported [31]. In the view of GPs, the rigidity of the DMP design excessively restricts freedom of action, for example in determining patient recall intervals or prescribing and treatment guidelines, and occasionally causes complications in the working routine of the practice [6]. The results also indicate that collaboration and interaction with specialist colleagues within the framework of DMPs is also perceived as unsatisfactory [35]. Consequently, although GPs believe that DMPs offer advantages in terms of patient care quality, they doubt that they have efficiency benefits for the healthcare system overall [37].

### Limitations and directions for future research

Although we were able to obtain a large heterogeneous random sample, it is necessary to mention various limitations of the survey. These include a regional recruitment concentration in three federal states and a limited response rate. It is also possible that more GPs who were interested in the subject took part in the survey (selection bias). It should also be noted that the survey was concerned with attitudes and experiences relating to DMP. This is no substitute for a specific intervention and accompanying studies evaluating the concrete benefit of individual programs and programe elements in everyday practice. Overall, there is still a need for independent studies looking at the extent to which DMPs improve the effectiveness of primary care [16].

## Conclusion

The results can be seen as validation that DMPs have arrived in primary healthcare and are endorsed by GPs as important instruments for the continuous and systematic care of chronically ill and multimorbid patients. Beyond this positive basic attitude, the respondents mentioned a series of weaknesses, which often obstruct the efficient and smooth coordination of DMPs within everyday clinical practice.

### Recommendations

Against this background, the following approaches were offered for optimising DMPs in line with the realities of primary healthcare:

- Documentation requirements should be examined and restricted. At the same time, interactions with the DMP datacentre and the (re)enrolment of patients in treatment programs should be significantly simplified and/or speeded up. Moreover, updates and modifications should be done in a way that avoids GPs having to deal with huge changeover problems in their time-critical practice routines.
- Allowing GPs greater freedom of action (setting recall intervals, drug treatment) is not in itself inconsistent with the DMP concept. Conversely, allowing GPs more (decision-making) flexibility would help to better address individual patient needs [32]. DMPs could also make provision for a greater degree of delegation.
- There seems to be a pressing need for better structuring of the interaction with other healthcare levels and for making this more effective. Only by solving the current interface problems will it be possible to sustainably achieve the declared goal of an interlinked supply chain [8, 42, 44, 46].
- The implementation of better incentive and remuneration structures can help to retain patients in the programs. A prerequisite for this is the provision of better information by the health insurance funds (continuous information, special offers to motivate patients).
- A broader and more differentiated offering of mandatory training courses would help to more accurately address the challenges faced by GPs in the everyday healthcare setting. In terms of reducing the burden on GPs and improving the efficiency of primary care, it would be useful to open training courses up to practice staff to a greater extent [35].
- In order to ensure greater GP compliance within DMPs, minimise practical supply problems and involve practices more effectively in the quality control process, programs should be evaluated and improved with greater involvement of GP experiences [7, 10, 11]. The same applies to the development of new DMPs. In fact, it is possible to observe an increased tendency to involve GPs, as is shown by the current process of revising the CHD and type 2 diabetes DMPs. General practitioners are systematically involved in this, and practical improvements have been made in terms of alignment with the guidelines and the practicability of treatment models [47, 48].
- And last but not least, consideration should be given to establishing a more effort-based fee structure for participating doctors, e.g. by creating more billing codes and greater recognition of doctors who participate in more than one DMP [42, 44, 46].

## Supplementary Information

Questionnaire: Disease management programs from a general practitioner’s point of view

## Abbreviations

DMP: Disease management program
GP: General practitioner

## References

[1] Fullerton B, Nolte E, Erler A (2011). Qualität der Versorgung chronisch Kranker in Deutschland [Quality of care of the chronically ill in Germany]. Z Evid Fortbild Qual Gesundhwes.

[2] Raghupathi W, Raghupathi V (2018). An empirical study of chronic diseases in the United States: a visual analytics approach to public health. Int J Environ Res Public Health.

[3] World Health Organization. The global burden of the chronic. 2019. https://www.who.int/nutrition/topics/2_background/en/. Accessed 11 Nov 2020.

[4] Bundesversicherungsamt. Zulassung der strukturierten Behandlungsprogramme [Approval of structured treatment programmes]. 2018. https://www.bundesversicherungsamt.de/weiteres/disease-management-programme/zulassung-disease-management-programme-dmp.html. Accessed 11 Nov 2020.

[5] Gemeinsamer Bundesausschuss [Federal Joint Committee, Innovation Committee].. Disease-management-programme [disease management programmes]. 2020. https://www.g-ba.de/themen/disease-management-programme/. Accessed 11 Nov 2020.

[6] Miksch A, Trieschmann J, Ose D (2011). DMP und Praxis: Stellungnahme von Hausärzten und Veränderung von Praxisabläufen zur Umsetzung des DMP Diabetes mellitus Typ 2 [General practitioners’ opinion and attitude towards DMPs and the change in practice routines to implement the DMP “diabetes mellitus type 2”]. Z Evid Fortbild Qual Gesundhwes.

[7] Gerlach FM, Szecsenyi J (2002). Warum sollen Disease-Management-Programme hausarztorientiert sein? Gründe, Grenzen und Herausforderungen [Why should Disease Management Programmes be oriented towards General practitioners? Reasons, limits and challenges. Dtsch Arztebl.

[8] Simcoe T, Catillon M, Gertler P (2019). Who benefits most in disease management programs: Improving target efficiency. Health Econ.

[9] Jutkowitz E, Nyman JA, Michaud TL, Abraham JM, Dowd B (2015). For what illnesses is a disease management program most effective?. J Occup Environ Med.

[10] Häussler B, Berger U (2004). Bedingungen für effektive Disease-Management-Programme [Conditions for effective Disease Management Programmes].

[11] Ahmed S, Ware P, Visca R (2015). The prevention and management of chronic disease in primary care: recommendations from a knowledge translation meeting. BMC Res Notes.

[12] Köhler Th, Leinert J, Südhoff S (2012). Ergebnisse der AOK-Bundesauswertungen zur gesetzlichen Evaluation der DMP für die Indikation Diabetes mellitus Typ 2 [Results of AOK Federal Association analyses for evaluating the Type 2 diabetes DMP]. Monit Versorgungsforsch.

[13] Szecsenyi J, Rosemann T, Joos S (2008). German diabetes disease management programs are appropriate for restructuring care according to the Chronic Care Model. An evaluation with the Patient Assessment of Chronic Illness Care instrument. Diabetes Care.

[14] Bundesversicherungsamt [Federal Insurance Office]. Tätigkeitsbericht 2017.. https://www.bundesversicherungsamt.de/fileadmin/redaktion/allgemeine_dokumente/2018BVA_Jahresbericht2017_web.pdf. Accessed 11 Nov 2020.

[15] Linder R (2011). Does DMP have an impact on quality?—An empirical study using routine data. Dtsch Med Wochenschr.

[16] Fuchs S, Henschke C, Blümel M (2014). Disease-Management-Programme für Diabetes mellitus Typ 2 in Deutschland. Abschätzung der Effektivität anhand einer systematischen Literaturübersicht [Disease Management Programmes for Type 2 diabetes in Germany. Estimate of effectiveness by means of a systematic literature review. Dtsch Arztebl Int.

[17] Linder R, Ahrens S, Köppel D (2011). Nutzen und Effizienz des Disease-Management-Programms Diabetes mellitus Typ 2 [Benefit and Efficiency of the Type 2 Diabetes Disease Management Programme]. Dtsch Arztebl Int.

[18] Ostermann H, Hoess V, Mueller M (2012). Efficiency of the Austrian disease management program for diabetes mellitus type 2: a historic cohort study based on health insurance provider’s routine data. BMC Public Health.

[19] Sönnichsen AC, Winkler H, Flamm M (2010). The effectiveness of the Austrian disease management programme for type 2 diabetes: a cluster-randomised controlled trial. BMC Fam Pract.

[20] Graf C, Ullrich W, Marschall U (2008). Nutzenbewertung der DMP Diabetes mellitus – Neue Erkenntnisse aus dem Vergleich von DMP-Teilnehmern und Nichtteilnehmern anhand von GKV-Routinedaten und einer Patientenbefragung [Benefit analysis of the diabetes DMP – New findings from the comparison of DMP participants and non-participants based on routine Health Insurance data and a patient survey. Gesundheits Sozialpolitik.

[21] Ose D, Wensing M, Szecsenyi J (2009). Impact of primary care-based disease management on the health related quality of life in patients with type 2 diabetes and co-morbidity. Diabetes Care.

[22] Miksch A, Laux G, Ose D (2010). Is there a survival benefit within a German primary care-based disease management program?. Am J Manag Care.

[23] Kanniess F, Krockenberger K, Oepen P (2020). Wirksamkeit von Disease-Management-Programmen für Asthma und COPD? Ergebnisse einer Querschnittstudie [Efficacy of Disease Management Programs Asthma and COPD? Results of a Cross-Sectional Study]. Pneumologie.

[24] Schulte T, Mund M, Hofmann L (2016). Pilotstudie zur Evaluation des DMP Koronare Herzkrankheit – Entwicklung einer Methodik und erste Ergebnisse [A pilot study to evaluate the DMP for coronary heart disease—Development of a methodology and first results. Z Evid Fortbild Qual Gesundhwes.

[25] Renders CM, Valk GD, Griffin S (2001). Interventions to improve the management of diabetes in primary care, outpatient, and community settings: a systematic review. Diabetes Care.

[26] Nolte E, Knai C, Hofmarcher M (2012). Overcoming fragmentation in health care: chronic care in Austria, Germany and The Netherlands. Health Econ Policy Law.

[27] Kruis AL, Smidt N, Assendelft WJ (2013). Integrated disease management interventions for patients with chronic obstructive pulmonary disease. Cochrane Database Syst Rev.

[28] Leibovici O-K, Freimark D, Freedman LS (2017). Disease management in the treatment of patients with chronic heart failure who have universal access to health care: a randomized controlled trial. BMC Med.

[29] Schneider A, Szecsenyi J (2002). Disease management programmes—opportunity or threat to GP identity?. Z Allg Med.

[30] Kaduszkiewicz H, van den Busche H (2003). Disease-Management-Programme – Erwartungen und Befürchtungen von Hausärzten und Patienten [Disease Management Programmes—Fears and expectations of GPs and patients.

[31] Bullmann C, Straub C (2006). DMP zwischen Anspruch und Wirklichkeit: Eigentlich sollte alles besser werden [Disease management programs between aspiration and reality. Actually, everything was meant to become much better]. Z Arztl Fortbild Qualitatssich.

[32] Schulze J (2004). Disease Management Programm Diabetes melitus Typ 2. Heftige Kritik der teilnehmenden Ärzte [Disease Management Programme for type 2 diabetes. Strong criticism from participating doctors]. Dtsch Arztebl.

[33] Graf C, Elkeles T, Kirschner W (2009). Is there a selection bias in disease management programmes for diabetes care? Results of a national insurance survey regarding DMP-participants and non-participants. Z Allg Med.

[34] Wangler J, Jansky M (2020). Anderthalb Dekaden Disease-Management-Programme – Eine Bilanz zum Status quo aus hausärztlicher Sicht [One and a Half Decades of Disease Management Programs—Status Quo From the Point of View of General practitioners]. Dtsch Med Wochenschr.

[35] Kaduszkiewicz H, van den Bussche H (2005). Auf den Zug aufgesprungen? Disease-Management-Programme aus der Perspektive von Hausärzten [Jumped on the bandwagon? Disease Management Programmes from the GP’s perspective. Disease-Management-Programme: Behandlung nach Maß? Jahrbuch für Kritische Medizin und Gesundheitswissenschaften.

[36] van Lente EJ. Mitmachen lohnt sich. [It’s worth taking part] Gesundheit und Gesellschaft SPEZIAL 10/2006, 9. Jahrgang.. https://www.aok-gesundheitspartner.de/imperia/md/gpp/bund/dmp/evaluation/befragung/gg_spezial_10_06_arztumfrage.pdf. Accessed 11 Nov 2020.

[37] Dickmann LM, Dickmann JR, Broocks A (2012). DMP depression? The family practitioner’s perspective. Z Allg Med.

[38] Faktorenanalyse FS, Baur N, Fromm S (2008). Factorial analysis. Datenanalyse mit SPSS für Fortgeschrittene. Ein Arbeitsbuch [Advanced data analysis with SPSS. A workbook].

[39] Mayring P (2010). Qualitative Inhaltsanalyse. Grundlagen und Techniken. [Qualitative Content Analysis. Basic principles and techniques].

[40] Cohen J (1988). Statistical power analysis for the behavioral sciences.

[41] Fernandez A, Grumbach K, Vranizan K (2001). Primary care physicians’ experience with disease management programs. J Gen Intern Med.

[42] Gerst T (2011). Disease-Management-Programme: Zehn Jahre DMP – Wenig Begeisterung [Disease Management Programmes: 10 years of DMP—Not much enthusiasm]. Dtsch Arztebl.

[43] Rieser S (2009). Disease-Management-Programme: ... und sie wirken doch! [Disease Management Programmes: … and they do work!]. Dtsch Arztebl.

[44] Weigeldt U (2014). Disease-Management-Programme: Unerwartete Bilanz nach zehn Jahren [Disease Management Programmes: Unexpected assessment after ten years]. Dtsch Arztebl.

[45] Willenborg P. 15 Jahre DMP: „Die Geschichte hat uns Recht gegeben“ [15 years of DMP: „History has proven us right“]. Ärzte Z.178:7.

[46] Egidi G, Werner S (2007). Disease Management Program Diabetes mellitus Type 2 in Bremen—Which Patients have Profited?. Z Allg Med.

[47] Deutsches Ärzteblatt. IQWiG empfiehlt Überarbeitung des DMP Koronare Herzkrankheit [IQWiG recommends revision of Coronary Heart Disease DMP]. 2018. https://www.aerzteblatt.de/nachrichten/92175/IQWiG-empfiehlt-Ueberarbeitung-des-DMP-Koronare-Herzkrankheit. Accessed 11 Nov 2020.

[48] Healy L, Ledwidge M, Gallagher J, Watson C, McDonald K (2019). Developing a disease management program for the improvement of heart failure outcomes: the do’s and the don’ts. Expert Rev Cardiovasc Ther.

