# Supplementary material for: Attitudes to and experience of disease management programs in primary care—an exploratory survey of general practitioners in Germany
Source: Wien Med Wochenschr. 2021 Aug 2;171(13-14):310–20. doi: 10.1007/s10354-021-00867-1 (PMC8484225; doi:10.1007/s10354-021-00867-1)
Supplement: Supplementary file 1 — Questionnaire: Disease management programs from a general practitioner’s point of view [file 10354_2021_867_MOESM1_ESM.docx]

**First of all, we would like to ask you a few general questions. Please answer these irrespective of whether you are currently participating in a DMP or not. This survey does not differentiate between individual healthcare levels.**

**1. In general terms: Do you basically consider Disease Management Programmes (DMP) to be a good thing or are you somewhat sceptical about them?**

⃝ Good thing ⃝ Somewhat sceptical ⃝ Undecided ⃝ Prefer not to say

**2. In your opinion or experience, how great is the overall benefit of DMPs for patient care?**

⃝ Very great ⃝ Fairly great ⃝ Fairly small ⃝ No benefit ⃝ Difficult to say, don't know

**3. Would you say your basic attitude to DMP has changed for the better or for the worse over the last few years?**

⃝ Improved significantly ⃝ Improved slightly ⃝ Remained the same

⃝ Worsened slightly ⃝ Worsened significantly

**4. Opinions differ as to the impact that DMP have had on primary patient care and on the healthcare system since they were introduced in 2003.**

**Which of the following statements do you agree with?**

| ***Disease Management Programmes have…*** | Completely agree | Mostly  agree | Mostly  disagree | Completely  disagree |
| --- | --- | --- | --- | --- |
| ... helped to ensure that patients are increasingly treated on the basis of evidence-based medicine and relevant guidelines | ⃝ | ⃝ | ⃝ | ⃝ |
| ... improved compliance of chronically ill patients due to better advice and support | ⃝ | ⃝ | ⃝ | ⃝ |
| ... restricted the therapeutic freedom of GPs | ⃝ | ⃝ | ⃝ | ⃝ |
| ... effectively improved the collaboration between GPs and consultants in the care of chronic diseases | ⃝ | ⃝ | ⃝ | ⃝ |
| ... resulted in patients being treated less individually, with less account being taken of their specific needs | ⃝ | ⃝ | ⃝ | ⃝ |
| ... strengthened the position of GPs in the care of chronically ill patients within the healthcare system | ⃝ | ⃝ | ⃝ | ⃝ |
| ... not significantly changed the quality of care for chronically ill patients | ⃝ | ⃝ | ⃝ | ⃝ |

| ***Disease Management Programmes have…*** | Completely agree | Mostly  agree | Mostly  disagree | Completely  disagree |
| --- | --- | --- | --- | --- |
| ... helped to prevent over or under-treatment (more efficient use of resources) | ⃝ | ⃝ | ⃝ | ⃝ |
| ... resulted in a great deal of unnecessary bureaucracy and/or documentation work | ⃝ | ⃝ | ⃝ | ⃝ |
| ... resulted in a clearly defined procedure in the care of the chronically ill, thereby increasing transparency of decision-making and ensuring the safety of medical action | ⃝ | ⃝ | ⃝ | ⃝ |
| ... brought about a change in workflows and responsibilities within the practice | ⃝ | ⃝ | ⃝ | ⃝ |
| ... helped to ensure that chronically ill patients are cared for proactively and continuously rather than merely reactively and in response to acute problems | ⃝ | ⃝ | ⃝ | ⃝ |
| ... resulted in successful (therapeutic) management of multi-morbid and chronically ill patient groups | ⃝ | ⃝ | ⃝ | ⃝ |
| ... increased the dependency of GPs upon Health Insurers | ⃝ | ⃝ | ⃝ | ⃝ |
| ... resulted in more efficient patient care (e.g. through limitation to key indicators, avoiding multiple documentation and duplicated treatments) | ⃝ | ⃝ | ⃝ | ⃝ |
| ... involved a heavy organisational/logistical burden for the practice managers | ⃝ | ⃝ | ⃝ | ⃝ |
| … improved the diagnostic and therapeutic safety of GPs | ⃝ | ⃝ | ⃝ | ⃝ |
| ... reduced costs for the healthcare system (in your opinion) | ⃝ | ⃝ | ⃝ | ⃝ |

**Please only answer the following questions if you are currently participating in one or more DMP or have previously done so. Otherwise, please skip directly to Question 16.**

**5. Are you participating in at least one DMP?**

⃝ Yes, am currently participating ⃝ Have previously participated but no longer

⃝ No **=> please skip directly to Question 16**

**6. Does or did your DMP participation relate to all or only certain Health Insurers?**

⃝ Relates to all HI ⃝ Only relates to certain HI ⃝ Partly one, partly the other

**7. In which of the following DMP are you currently participating or have previously participated?**

Bronchial asthma

⃝ Am currently participating ⃝ Have previously participated ⃝ Am not participating

Coronary heart disease (CHD)

⃝ Am currently participating ⃝ Have previously participated ⃝ Am not participating

COPD

⃝ Am currently participating ⃝ Have previously participated ⃝ Am not participating

Type 1 diabetes

⃝ Am currently participating ⃝ Have previously participated ⃝ Am not participating

Type 2 diabetes

⃝ Am currently participating ⃝ Have previously participated ⃝ Am not participating

**8. From your own experience, how would you rate the DMP in which you are currently participating or have previously participated?**

Bronchial asthma
⃝ Very good ⃝ Fairly good ⃝ Fairly poor ⃝ Very poor ⃝ Have not participated

Coronary heart disease (CHD)
⃝ Very good ⃝ Fairly good ⃝ Fairly poor ⃝ Very poor ⃝ Have not participated

COPD
⃝ Very good ⃝ Fairly good ⃝ Fairly poor ⃝ Very poor ⃝ Have not participated

Type 1 diabetes
⃝ Very good ⃝ Fairly good ⃝ Fairly poor ⃝ Very poor ⃝ Have not participated

Type 2 diabetes
⃝ Very good ⃝ Fairly good ⃝ Fairly poor ⃝ Very poor ⃝ Have not participated

**9. How would you rate the following elements of DMP?**

Mandatory training courses for participating doctors

⃝ Very good ⃝ Fairly good ⃝ Fairly poor ⃝ Very poor ⃝ Don't know

Structured training courses for enrolled patients to support their treatment

⃝ Very good ⃝ Fairly good ⃝ Fairly poor ⃝ Very poor ⃝ Don't know

Documentation (including treatment steps, investigation and treatment results)

⃝ Very good ⃝ Fairly good ⃝ Fairly poor ⃝ Very poor ⃝ Don't know

External recording of the treatment (including checking documentation for plausibility and completeness, evaluation)

⃝ Very good ⃝ Fairly good ⃝ Fairly poor ⃝ Very poor ⃝ Don't know

Regular recall of patients

⃝ Very good ⃝ Fairly good ⃝ Fairly poor ⃝ Very poor ⃝ Don't know

**10. With regard to the DMP in which you have so far participated, what aspects did you like and what aspects do you think are worth highlighting as particularly positive? A few key points will suffice.**

__________________________________________________________________________________

__________________________________________________________________________________

__________________________________________________________________________________

**11. And, with regard to the DMP in which you have so far participated, what aspects did you not like and what aspects did you perceive as negative?**

__________________________________________________________________________________

__________________________________________________________________________________

__________________________________________________________________________________

**12. How frequently did you encounter obstacles or complications in your daily practice as a result of your participation in one or more DMP?** (By this we mean e.g. delays or difficulties in the working routine of the practice)

⃝ Frequently ⃝ Occasionally ⃝ Rarely ⃝ Never

**13. How would you rate overall the extent to which the treatment of enrolled patients benefited from the DMP in which you have so far participated?**

⃝ Very much ⃝ Quite a lot ⃝ Not so much ⃝ Not at all ⃝ It differs a lot, difficult to say

**14. Have you trained members of your practice staff as a result of your DMP participation?**

⃝ Yes, one person ⃝ Yes, more than one person up to half the staff ⃝ Yes, more than half the staff ⃝ Yes, the entire staff ⃝ No, not trained anyone

**15. From your own experience of DMP, which of the following statements do you agree with?**

|  | Completely agree | Mostly  agree | Mostly  disagree | Completely  disagree |
| --- | --- | --- | --- | --- |
| "I have learnt something new about diagnosis and/or treatment through participating in Disease Management Programmes." | ⃝ | ⃝ | ⃝ | ⃝ |
| "I essentially follow the DMP recommendations for (drug) treatment." | ⃝ | ⃝ | ⃝ | ⃝ |
| "I can hardly imagine doing without Disease Management Programmes in my practice." | ⃝ | ⃝ | ⃝ | ⃝ |
| "I have improved my own skills as a result of participating in Disease Management Programmes." | ⃝ | ⃝ | ⃝ | ⃝ |
| "The advantages of Disease Management Programmes outweigh the disadvantages and difficulties." | ⃝ | ⃝ | ⃝ | ⃝ |

**16. There are currently four new DMP in the development and/or implementation phase. In which of these might you consider participating or definitely intend to participate?**

Heart failure

⃝ Intend to participate ⃝ Might consider it ⃝ Definitely not ⃝ Don't know yet

Chronic back pain

⃝ Intend to participate ⃝ Might consider it ⃝ Definitely not ⃝ Don't know yet

Depression

⃝ Intend to participate ⃝ Might consider it ⃝ Definitely not ⃝ Don't know yet

Osteoporosis

⃝ Intend to participate ⃝ Might consider it ⃝ Definitely not ⃝ Don't know yet

Rheumatoid arthritis

⃝ Intend to participate ⃝ Might consider it ⃝ Definitely not ⃝ Don't know yet

**17. What improvements would you like to see in Disease Management Programmes in future?**

__________________________________________________________________________________

__________________________________________________________________________________

__________________________________________________________________________________

**18. In your view, how important is it for GPs to be more involved than before in the development of new DMP and the improvement of existing DMP?**

⃝ Very important ⃝ Fairly important ⃝ Fairly unimportant ⃝ Difficult to say, don't know

*Finally, we need some statistical data from you. As with the rest of the questionnaire, this information will be treated in strict confidence and anonymised.*

**You are…**

⃝ Male ⃝ Female ⃝ Other

Your **age**: _____

**Where is your practice located?** In a community/town with...

⃝ more than 100,000 ⃝ 20,000 to 100,000 ⃝ 5,000 to 20,000 ⃝ less than 5,000 inhabitants

**Which structural model best describes your practice?**

⃝ Single-handed practice (you are the only doctor) ⃝ Single-handed practice with salaried doctors

⃝ Group practice ⃝ Medical Centre ⃝ Other

**How many patients** does your practice treat per quarter?

⃝ 500 - 750 ⃝ 751 - 1000 ⃝ 1001 - 1500 ⃝ More than 1500

**Do you h additional training in diabetology?**

**Thank you for taking part!**

**If you have completed this survey in writing, please use**

**the return envelope provided.**

Is there anything else you would like to tell us?

You can record your suggestions, comments or criticisms here.

__________________________________________________________________________

__________________________________________________________________________

__________________________________________________________________________
